# Supplementary material for: Colorectal cancers with distinct metastatic potential trigger divergent early T cell responses
Source: bioRxiv. 2026 Jul 4:2026.06.30.735606. Preprint. [Version 1] doi: 10.64898/2026.06.30.735606 (PMC13344958; doi:10.64898/2026.06.30.735606)
Supplement: Supplement 2 [file NIHPP2026.06.30.735606v1-supplement-2.pdf]

bioRxiv preprint doi: https://doi.org/10.64898/2026.06.30.735606; this version posted July 4, 2026. The copyright holder for this preprint (which was not certified by peer review) is the author/funder, who has granted bioRxiv a license to display the preprint in perpetuity. It is made available under a CC-BY 4.0 International license.

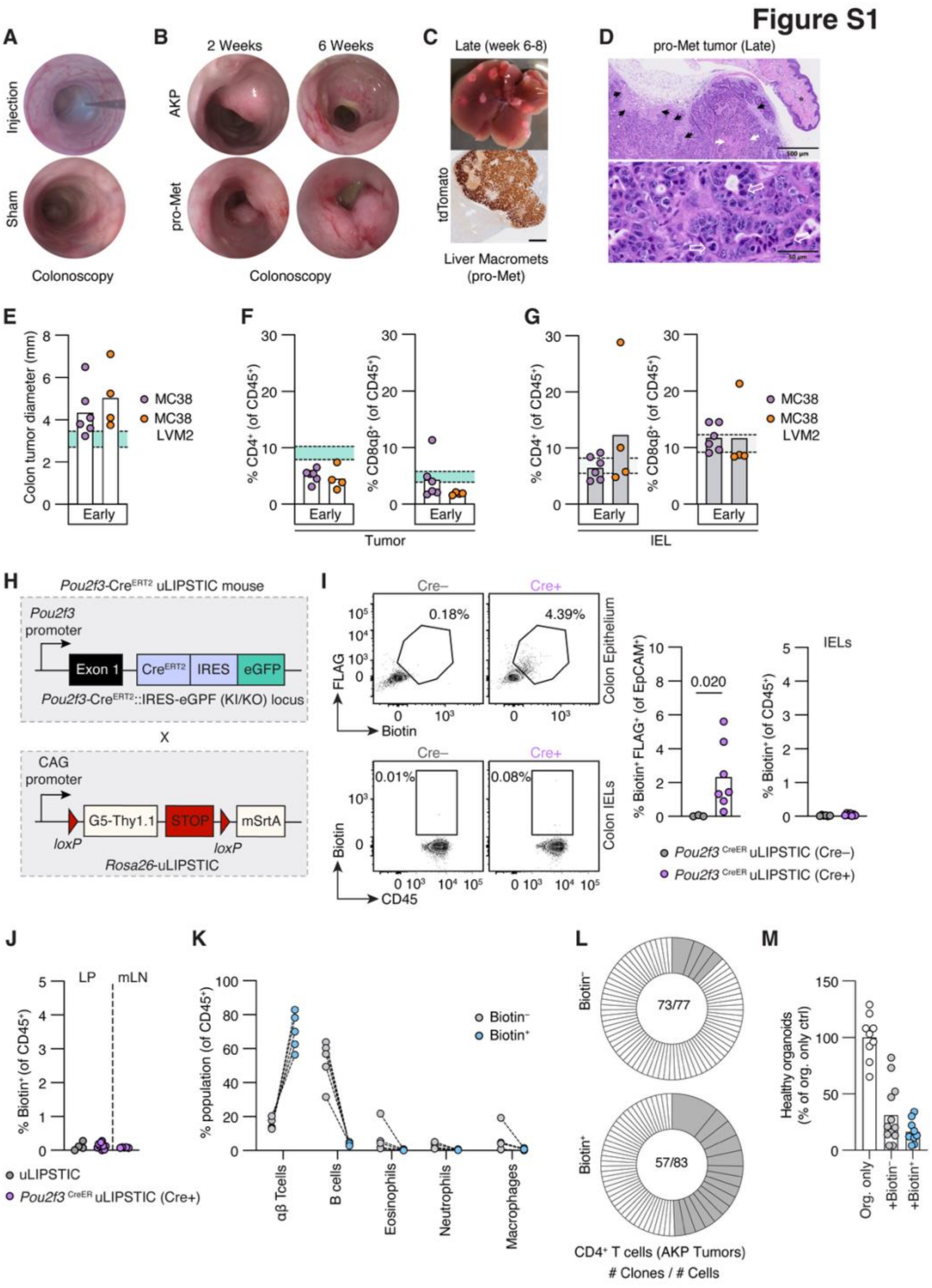

Figure S1: Extended data related to Figures 1 and 2.

(A) Colonoscopy images of injection with needle in view (top) and sham control two weeks post-injection (bottom).

bioRxiv preprint doi: https://doi.org/10.64898/2026.06.30.735606; this version posted July 4, 2026. The copyright holder for this preprint (which was not certified by peer review) is the author/funder, who has granted bioRxiv a license to display the preprint in perpetuity. It is made available under a CC-BY 4.0 International license.

(B) Colonoscopy images of tumor growth in AKP and pro-Met CRC models at 2 and 6 weeks post-tumor injection.

(C) Liver macrometastases (top) and tdTomato immunohistochemistry (bottom) from liver sections in the pro-Met CRC model at 6-8 weeks post tumor injection in the colon. Scale bar = 500 µm.

(D) Representative H&E-stained section of a late-stage pro-Met adenocarcinoma expanding into the underlying connective tissue of the rectum (asterisk) and distal colon. The adenocarcinoma (top image) exhibited multifocal areas of necrosis (white arrows) and desmoplasia (reactive fibrous connective; black arrows). High magnification (bottom image) showed the adenocarcinoma is composed by neoplastic columnar to polygonal epithelial cells with small to medium eosinophilic cytoplasm and round to oval nuclei containing one to two prominent nucleoli. Mitotic figures are present (open white arrows). Scale bar = 500 µm for top image, and = 50 µm for bottom image.

(E) Colon tumor diameter 2 weeks after the MC38 and MC38 LVM2 tumor injections. Each symbol represents a mouse. Shaded area represents the 95% confidence interval of colon tumor diameter of AKP tumors at the early (2-week) stage.

(F) Frequencies of $CD4^+$ and $CD8\alpha\beta^+$ T cells among $CD45^+$ T cells in colon tumors of respective tumor lines at the early CRC stage. Shaded area represents the 95% confidence interval of T cell populations in AKP tumors at the early stage.

(G) Frequencies of $CD4^+$ and $CD8\alpha\beta^+$ T cells among $CD45^+$ IEL isolated from colon epithelium of tumor-injected mice at the early CRC stage. Shaded area represents the 95% confidence interval of T cell populations in Sham IEL.

(H) Schematic diagram showing the genetics of the *Pou2f3*^{CreERT2} uLIPSTIC model.

(I) *Pou2f3*^{CreER} uLIPSTIC mice (either Cre– or Cre+) were treated by oral gavage with 200 µL of 50 mg/mL tamoxifen on days -3 and -1. On day 0, six doses of biotin-LPETG substrate were injected intraperitoneally over a course of 2 h (8 µmol/1st dose, followed by 2 µmol for each subsequent dose). Mice were sacrificed for flow cytometry analysis 1 h after the last substrate injection. Dot plots and frequency of FLAG and biotin expression in colonic epithelial cells (top). Dot plots and frequency of biotin expression in $CD45^+$ cells harvested from colon epithelium (bottom).

(J) Frequency of $biotin^+$ among $CD45^+$ cells isolated from colon lamina propria (LP) and mesenteric lymph nodes (mLN) of AKP^{SrtA}-injected mice following biotin-LPETG substrate administration.

(K) Frequencies of indicated immune cell populations among $biotin^-$ and $biotin^+$ $CD45^+$ cells.

(L) Pie charts showing $biotin^-$ (top) and $biotin^+$ (bottom) $CD4^+$ T cell clones isolated from AKP^{SrtA} tumors two weeks post transplantation in *Pou2f3*^{CreER} uLIPSTIC mouse. Grey sections represent expanded clones (detected more than twice), and white sections represent singlet clones. Number inside chart

bioRxiv preprint doi: https://doi.org/10.64898/2026.06.30.735606; this version posted July 4, 2026. The copyright holder for this preprint (which was not certified by peer review) is the author/funder, who has granted bioRxiv a license to display the preprint in perpetuity. It is made available under a CC-BY 4.0 International license.

represents the number of $CD4^+$ T cell clones / number of recovered $CD4^+$ T cells. Each pie chart represents one mouse.

(M) Healthy organoid frequency calculated by normalizing the number of healthy organoids recovered per well to the average number of healthy organoids recovered in control (organoids only).

bioRxiv preprint doi: https://doi.org/10.64898/2026.06.30.735606; this version posted July 4, 2026. The copyright holder for this preprint (which was not certified by peer review) is the author/funder, who has granted bioRxiv a license to display the preprint in perpetuity. It is made available under a CC-BY 4.0 International license.

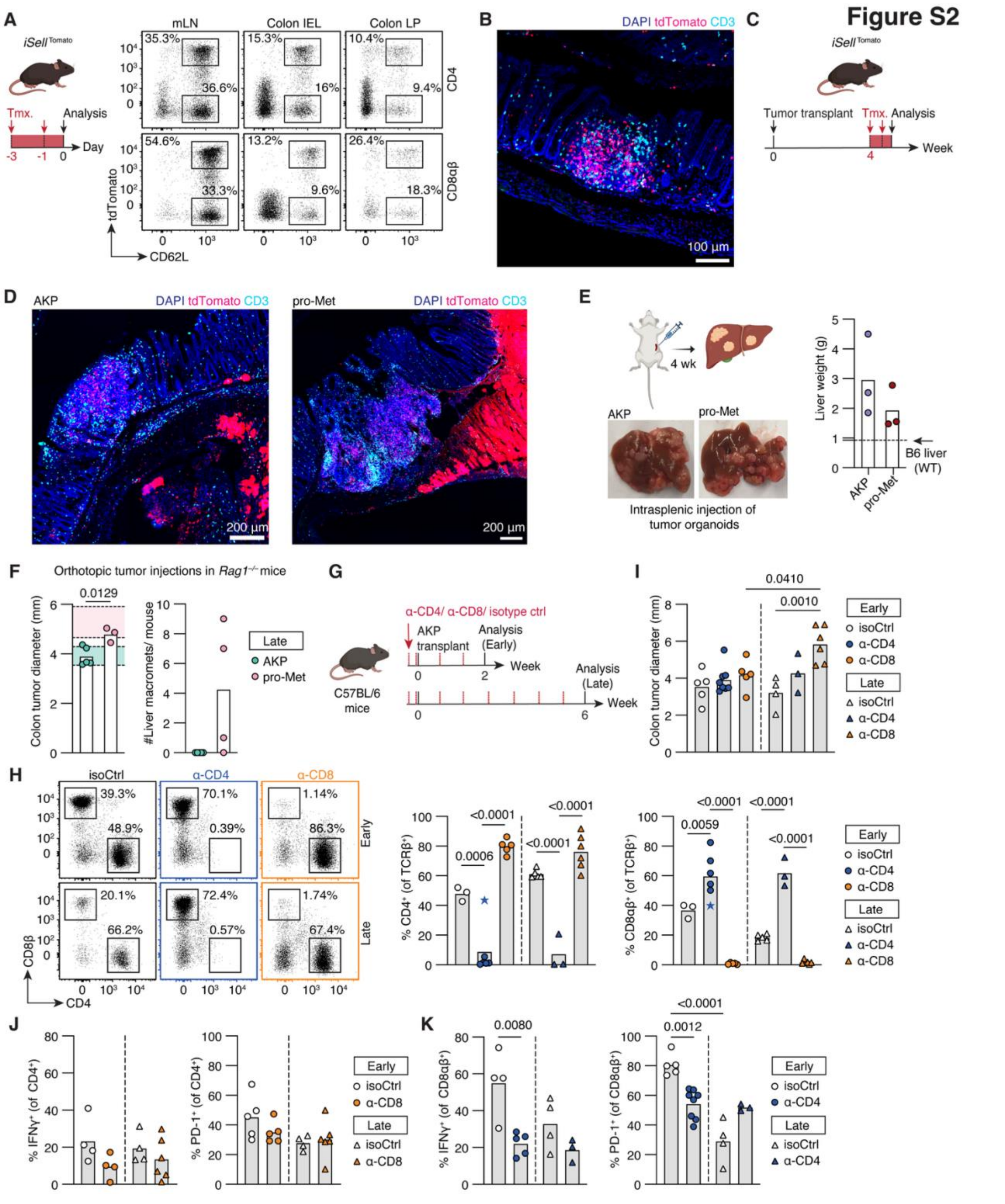

Figure S2: Effects of modulating the adaptive immune system on AKP tumor outcome, and extended controls for Figure 3.

bioRxiv preprint doi: https://doi.org/10.64898/2026.06.30.735606; this version posted July 4, 2026. The copyright holder for this preprint (which was not certified by peer review) is the author/funder, who has granted bioRxiv a license to display the preprint in perpetuity. It is made available under a CC-BY 4.0 International license.

(A) Experimental design (left) and dot plot (right) showing CD62L^{+} Tomato^{+} and Tomato^{-} among CD4^{+} (top) and CD8αβ^{+} (bottom) T cells across the mLN, colon IEL and LP 1 day after the second dose of tamoxifen administration in i*Sell*^{Tomato} mice.

(B) Immunofluorescence (IF) staining of mouse colon harvested 1 day after the second dose of tamoxifen administration in i*Sell*^{Tomato} mice. Staining shows DAPI, tdTomato, and CD3. Magnification= 20X. Images are representative of n=2 mice.

(C) Experimental design. i*Sell*^{Tomato} mice were transplanted with AKP or pro-Met organoids, and treated with tamoxifen 4 weeks later. Colons were isolated for IF staining one day after the second tamoxifen dose.

(D) IF staining of colons isolated from (C). tdTomato represents both tumor cells (bright on the right of the images), and also tdTomato^{+} immune cells. Images are representative of n=2 mice/ condition.

(E) Scheme of the intrasplenic injection model and display of AKP and pro-Met liver tumor growth. Quantification of tumor load by liver weight. The dashed line indicates average healthy adult B6 liver weight[77].

(F) *Rag1*$^{-/-}$ mice were orthotopically injected with AKP or pro-Met tumor organoids using a colonoscope. Average colon tumor diameter per mouse (left) and number of liver macrometastases (right) were assessed 6-7 weeks after tumor injection. Shaded areas represent the 95% confidence interval of colon tumor size in AKP (green) and pro-Met (pink) tumor organoids injected into immunocompetent SPF mice.

(G) Experimental design for panels H–K. C57BL/6 mice were injected with 200 μg/mouse anti-CD4 (α-CD4), anti-CD8 (α-CD8), or isotype control (isoCtrl) two days before orthotopic AKP tumor organoid injection. Antibody injections were repeated on the day of tumor injection and every 5-7 days. The dashed red line indicates antibody injections. Mice were sacrificed for analysis 2 or 6 weeks after tumor injection.

(H) Dot plots and frequencies of CD4^{+} (middle) and CD8β^{+} (right) among TCRβ^{+} T cells at the indicated conditions and time points. The blue star symbol refers to a mouse with unsuccessful CD4 T cell depletion and hence was excluded from further analysis.

(I) Average colon tumor diameter per mouse under the indicated treatment conditions and analysis time points.

(J) Frequency of IFN-γ^{+} (left) and PD-1^{+} (right) among CD4^{+} T cells isolated from colon tumors following *ex vivo* PMA/ionomycin stimulation .

(K) Frequency of IFN-γ^{+} (left) and PD-1^{+} (right) among CD8αβ^{+} T cells isolated from colon tumors following *ex vivo* PMA/ionomycin stimulation.

Unpaired Student's t-tests were performed for (E) and (F). Ordinary one-way ANOVA with Tukey's multiple comparison tests were performed in (I–K).

bioRxiv preprint doi: https://doi.org/10.64898/2026.06.30.735606; this version posted July 4, 2026. The copyright holder for this preprint (which was not certified by peer review) is the author/funder, who has granted bioRxiv a license to display the preprint in perpetuity. It is made available under a CC-BY 4.0 International license.

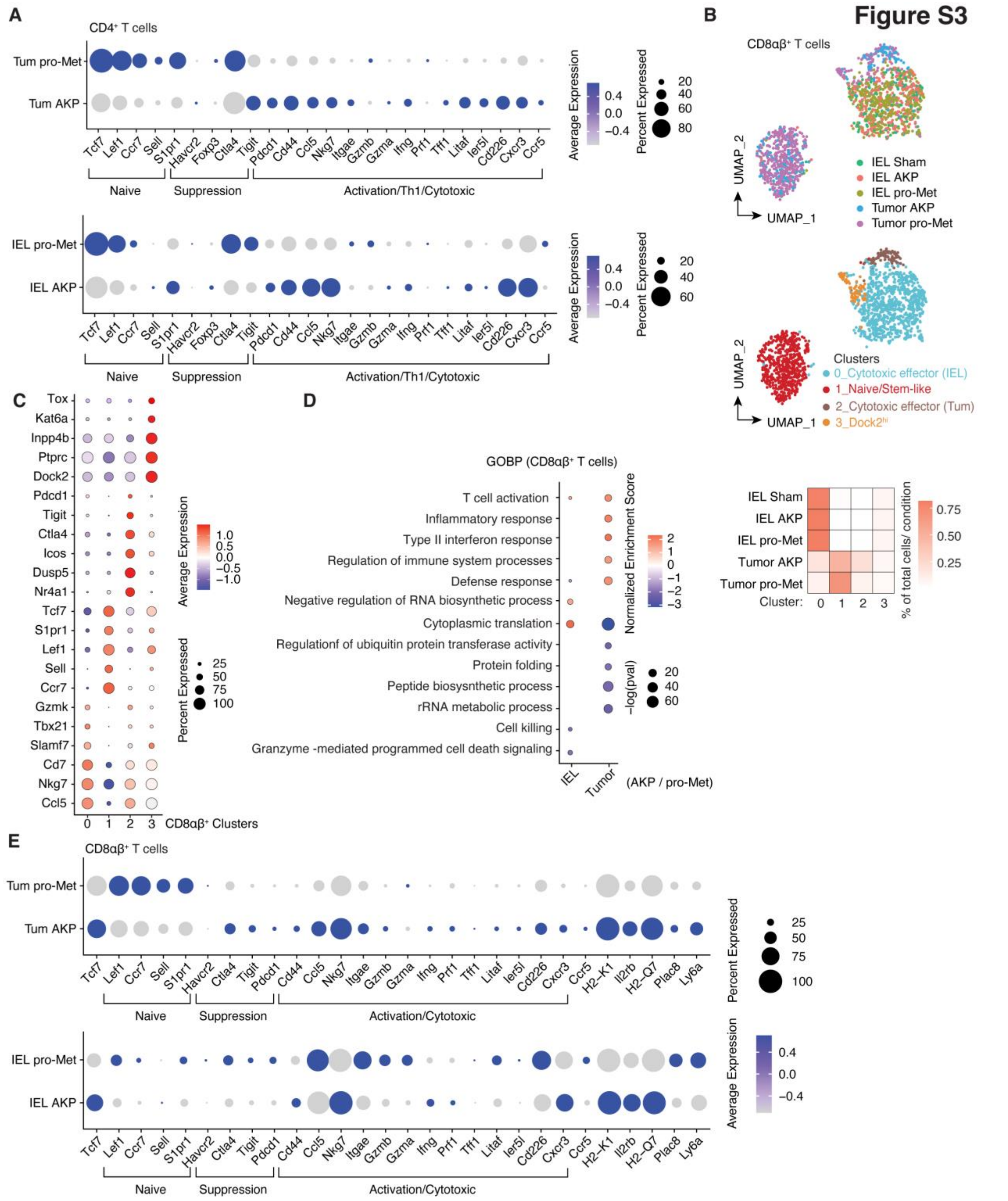

bioRxiv preprint doi: https://doi.org/10.64898/2026.06.30.735606; this version posted July 4, 2026. The copyright holder for this preprint (which was not certified by peer review) is the author/funder, who has granted bioRxiv a license to display the preprint in perpetuity. It is made available under a CC-BY 4.0 International license.

Figure S3: Transcriptional profiles of recently recruited CD4⁺ and CD8αβ⁺ T cells during the early CRC stages. Extended data for Figure 4.

i*Sell*^{Tomato} mice orthotopically injected with sham, AKP, or pro-Met tumors using colonoscope 1 day post second tamoxifen dose. CD62L^{-} Tomato+ TCRαβ^{+} T cells from colon IEL and tumor were single-cell RNA sequenced. CD4^{+} and CD8αβ^{+} T cells were separated for further analysis.

(A) Dot plot of selected differentially expressed genes between AKP and pro-Met tumor-derived CD4^{+} T cells (top) or AKP IEL and pro-Met IEL-derived CD4^{+} T cells (bottom).

(B) UMAP of CD8αβ^{+} T cells visualizing cells isolated per condition (top), main identified clusters using Seurat (SCT normalization, resolution = 0.3) (middle), and a heat map (bottom) showing the frequency of cells per condition across clusters. Each condition (row) represents 100%.

(C) Dot plot of CD8αβ^{+} T cell clusters visualized in (B), showing the selected markers expressed per cluster.

(D) Selected Gene Ontology Biological Processes (GOBP) differentially expressed between AKP, pro-Met IEL and tumor-derived CD8αβ^{+} T cells. Colors represent the Normalized Enrichment Score (NES) associated with that pathway in IEL and Tumor and the dot radius represents -log of the adjusted p-value of that enrichment.

(E) Dot plot of selected differentially expressed genes between AKP and pro-Met tumor-derived CD8αβ^{+} T cells (top) or AKP IEL and pro-Met IEL-derived CD8αβ^{+} T cells (bottom).

bioRxiv preprint doi: https://doi.org/10.64898/2026.06.30.735606; this version posted July 4, 2026. The copyright holder for this preprint (which was not certified by peer review) is the author/funder, who has granted bioRxiv a license to display the preprint in perpetuity. It is made available under a CC-BY 4.0 International license.

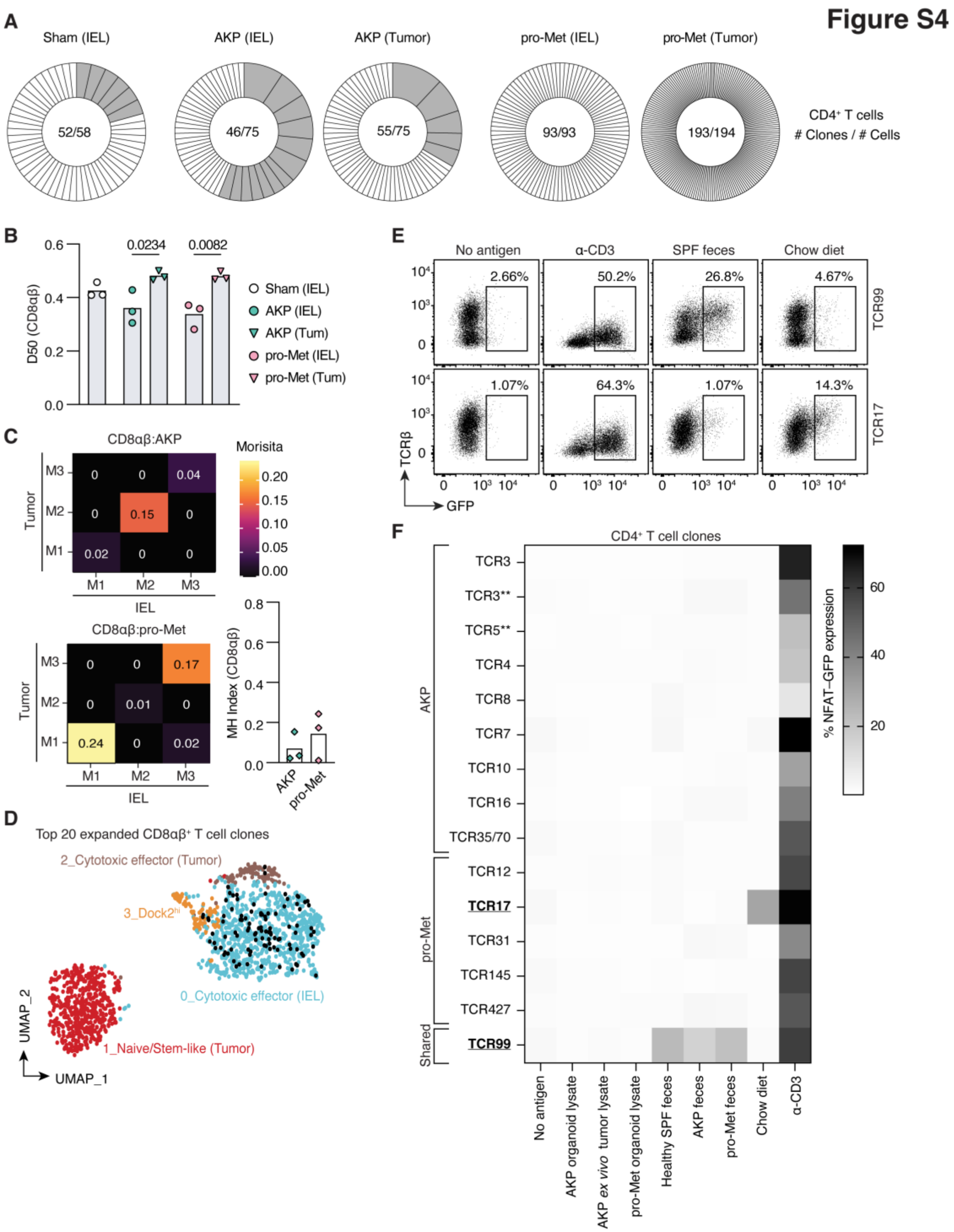

Figure S4: Minimal clonal expansion and clonal overlap of CD8αβ⁺ T cells across conditions, and extended data related to Figure 6.

(A) Pie charts showing CD62L⁻ Tomato⁺ CD4⁺ T cell clones isolated from i*Sell*^{Tomato} mice (IEL and Tumor) two weeks post tumor transplantation. Grey sections represent expanded clones (detected more than

bioRxiv preprint doi: https://doi.org/10.64898/2026.06.30.735606; this version posted July 4, 2026. The copyright holder for this preprint (which was not certified by peer review) is the author/funder, who has granted bioRxiv a license to display the preprint in perpetuity. It is made available under a CC-BY 4.0 International license.

twice), and white sections represent singlet clones. Number inside chart represents the number of $CD4^+$ T cell clones / number of recovered $CD4^+$ T cells. Each pie chart represents one mouse.

(B) Diversity index 50 (D50) of CD8αβ$^+$ T cell clones isolated from the indicated tissues and conditions.

(C) CD8αβ$^+$ T cell clonal sharing between IEL and tumor in AKP (top) and pro-Met (bottom) injected mice, represented by the MH index. M1,2,3 = Mouse 1,2,3.

(D) Top 20 expanded CD8αβ$^+$ T cell clones (black) overlayed on CD8αβ UMAP. Seurat-identified clusters (resolution =0.3) were visualized and labelled on the UMAP.

(E–F) TCRs from expanded $CD4^+$ T cell clones were reconstructed and expressed in TCR-deficient murine NFAT-GFP T cell hybridomas and co-cultured with antigen-pulsed splenic dendritic cells (DCs) for 18–40 h. NFAT-GFP expression indicating TCR reactivity is represented by dot plots in (E) and summarized in the heatmap in (F) for cloned NFAT hybridoma cell lines with high TCRβ expression. Frequencies indicate GFP expression out of $CD4^+$ T cells in response to no antigen stimulation, anti-CD3 treatment, or stimulation with tumor, fecal, or dietary proteins. AKP and pro-Met organoid lysates were prepared from *in vitro* cultured organoids. AKP *ex vivo* tumor lysate was prepared from AKP tumors collected 2 weeks after orthotopic tumor injections. AKP and pro-Met mouse fecal lysates were collected from early-stage AKP or pro-Met injected mice.

Cloned TCR sequences are listed in Supplementary Table 1. Underlined TCR clones indicate clones with detected reactivities to broad proteins screened in this panel.

Ordinary one-way ANOVA with Tukey's multiple comparison test was performed in (B).

bioRxiv preprint doi: https://doi.org/10.64898/2026.06.30.735606; this version posted July 4, 2026. The copyright holder for this preprint (which was not certified by peer review) is the author/funder, who has granted bioRxiv a license to display the preprint in perpetuity. It is made available under a CC-BY 4.0 International license.

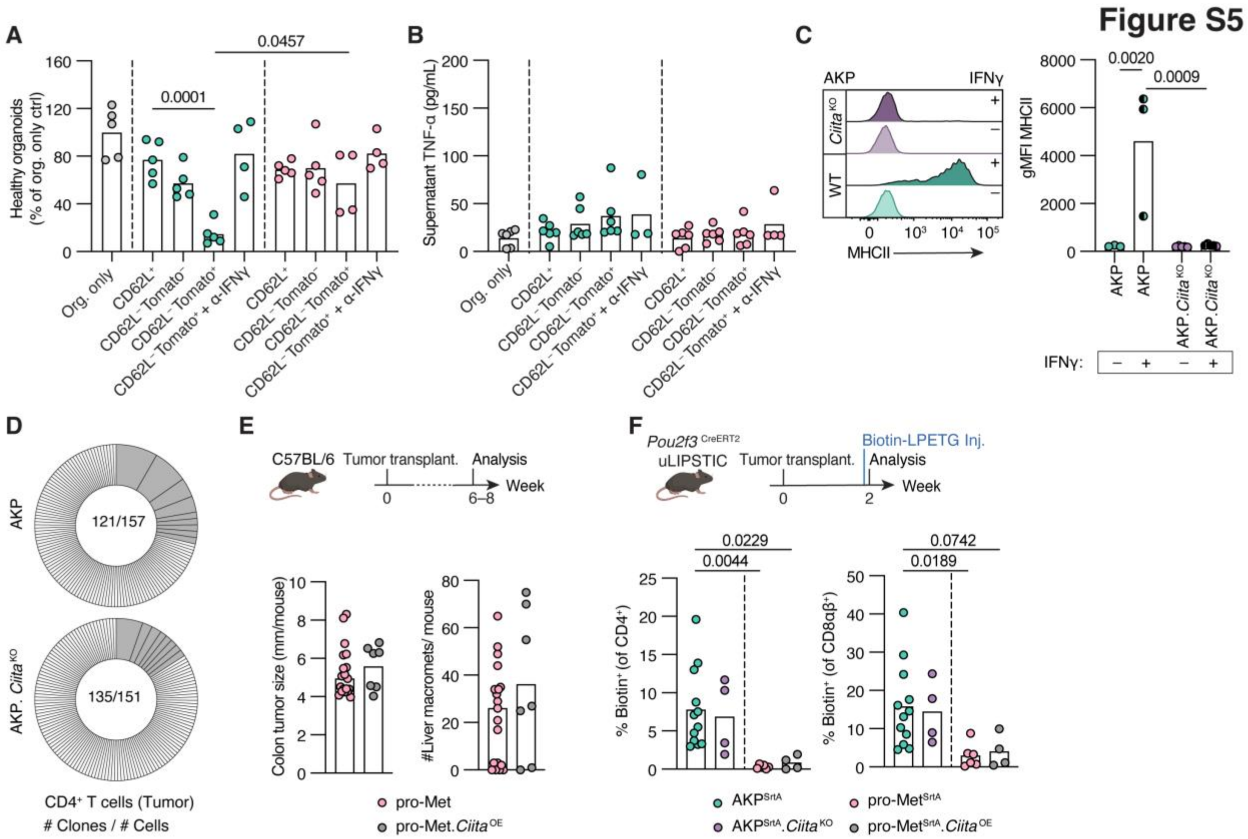

Figure S5: Extended data related to Figures 7 and 8.

(A) Healthy organoid frequency calculated by normalizing the number of healthy organoids recovered per well to the average number of healthy organoids recovered in control (organoids only).

(B) CBA analysis of co-culture supernatant collected on day 5. Graph shows levels of TNF-α per condition.

(C) MHCI gMFI of AKP and AKP.*Ciita* ^{KO} organoids in the presence or absence of IFN-γ stimulation.

(D) Pie charts showing CD62L^{-} Tomato^{+} CD4^{+} T cell clones isolated from i*Sell*^{Tomato} mice (IEL and Tumor) two weeks post AKP and AKP.*Ciita* ^{KO} tumor transplantation. Grey sections represent expanded clones (detected more than twice), and white sections represent clones detected once. Number inside chart represents the number of CD4^{+} T cell clones / number of recovered CD4^{+} T cells. Each pie chart represents one mouse.

(E) Average colon tumor size per mouse (left), and liver macrometastases (right) at the late tumor stage (6-8 weeks post transplantation). Pro-Met control shown in (E) is from the same dataset in Figures 1B and 1C. Ordinary one-way ANOVA with Tukey's multiple comparison test was performed for colon tumor size with all groups combined.

bioRxiv preprint doi: https://doi.org/10.64898/2026.06.30.735606; this version posted July 4, 2026. The copyright holder for this preprint (which was not certified by peer review) is the author/funder, who has granted bioRxiv a license to display the preprint in perpetuity. It is made available under a CC-BY 4.0 International license.

(F) Frequencies of biotin$^+$ among CD4$^+$ (left) and CD8αβ$^+$ (right) T cells harvested from the indicated tumors. Data from AKP^{SrtA} and pro-Met^{SrtA} is the same as in Figure 2C. Ordinary one-way ANOVA with Tukey's multiple comparison test was performed with all groups combined.

Supplementary Table 1: TCR used for NFAT-GFP hybridomas, extracted from indicated conditions and tissues.

TCR ID	Clone ID	Condition	Tissue	TRA CDR3	TRB CDR3	TRAV	TRAJ	TRBV	TRBJ	TRBD
TCR1	TRA_TRB_8.1	AKP	Tumor + IEL	CAPLNSAGNKLTF	CASSPRQFSNERLFF	TRAV13N-2	TRAJ17	TRBV3	TRBJ1-4	na
TCR1**	TRA_TRB_TRB_1.1	AKP	Tumor + IEL	CAPLNSAGNKLTF	CARTGGDNQDTQYF	TRAV13N-2	TRAJ17	TRBV13-3	TRBJ2-5	na
TCR3	TRA_TRB_41.1	AKP	Tumor	CAIEMSNYNVLYF	CASRDWTSAETLYF	TRAV13N-2	TRAJ21	TRBV10	TRBJ2-3	na
TCR3**	TRA_TRA_TRB_5.1	AKP	Tumor + IEL	CAVSAGSGYNKLTF	CASRDWTSAETLYF	TRAV3N-3	TRAJ11	TRBV11	TRBJ2-4	na
TCR4	TRA_TRA_TRB_6.1	AKP	Tumor + IEL	CALSGNSNNRIFF	CASSQEGQGYAEQFF	TRAV6N-6	TRAJ31	TRBV2	TRBJ2-1	na
TCR4**	TRA_TRA_TRB_6.1	AKP	Tumor + IEL	CALERPGTGSNRLTF	CASSQEGQGYAEQFF	TRAV13-1	TRAJ28	TRBV2	TRBJ2-2	na
TCR5	TRA_TRA_TRB_1.1	AKP	Tumor + IEL	CAVDYSNNRLTL	CASSPRQFSNERLFF	TRAV3N-3	TRAJ7	TRBV3	TRBJ1-4	na
TCR5**	TRA_TRA_TRB_1.1	AKP	Tumor + IEL	CALLNSAGNKLTF	CASSPRQFSNERLFF	TRAV6-5	TRAJ17	TRBV3	TRBJ1-4	na
TCR7	TRA_TRB_4.1	AKP	Tumor + IEL	CALLNTGKLIF	CASSQEGQGYAEQFF	TRAV12D-3	TRAJ37	TRBV2	TRBJ2-1	na
TCR8	TRA_TRB_5.1	AKP	Tumor + IEL	CALDYGSSGNKLIF	CASSPRQFSNERLFF	TRAV6-7-DV9	TRAJ32	TRBV3	TRBJ1-4	na
TCR10	TRA_TRB_6.1	AKP	Tumor + IEL	CALRNSAGNKLTF	CASSPRQFSNERLFF	TRAV12-1	TRAJ17	TRBV3	TRBJ1-4	na
TCR11	TRA_TRB_3.1	AKP	Tumor + IEL	CAPSNSAGNKLTF	CASSLRQFSNERLFF	TRAV7N-6	TRAJ17	TRBV4	TRBJ1-4	na
TCR13	TRA_TRB_10.1	AKP	Tumor	CAAANSAGNKLTF	CASSPRQFSNERLFF	TRAV7-3	TRAJ17	TRBV3	TRBJ1-4	na
TCR16	TRA_TRB_12.1	AKP	Tumor + IEL	CAMVNSAGNKLTF	CASSPRQFSNERLFF	TRAV13N-1	TRAJ17	TRBV3	TRBJ1-4	na
TCR35/70	TRA_TRB_30.1 (and TRA_TRB_24.1)	AKP	Tumor + IEL	CAASNSAGNKLTF	CASSPRQFSNERLFF	TRAV10	TRAJ17	TRBV3	TRBJ1-4	na
TCR12	TRA_TRB_7.1	pro-Met	Tumor + IEL	CAVSMPNTNKVVF	CASSETGIAETLYF	TRAV7-5	TRAJ34	TRBV13-1	TRBJ2-3	na
TCR17	TRA_TRB_13.1	pro-Met	Tumor + IEL	CVLGDPGSSGNKLIF	CASSLGGNQDTQYF	TRAV6-2	TRAJ32	TRBV3	TRBJ2-5	na
TCR31	TRA_TRB_31.1	pro-Met	Tumor	CAIWTGGYKVVF	CGARDRGNSDYTF	TRAV13N-1	TRAJ12	TRBV20	TRBJ1-2	na
TCR145	TRA_TRB_112.1	pro-Met	Tumor	CAARGSNYQLIW	CASSQDGVNQDTQYF	TRAV7-4	TRAJ33	TRBV5	TRBJ2-5	na
TCR427	TRA_TRB_64.1	pro-Met	Tumor	CAIGYNQGKLIF	CASSQDGVNQDTQYF	TRAV13N-2	TRAJ23	TRBV5	TRBJ2-5	na
TCR99	TRA_TRB_63.1 (and TRA_TRB_63.2)	AKP and pro-Met	Tumor + IEL	CAARAGYQNFYF	CASGDATGNTGQLYF	TRAV7D-2	TRAJ49	TRBV12-2+TRBV13-2	TRBJ2-2	TRBD1
